# Simultaneous occurrence of splenic diffuse large B cell lymphoma and gastrointestinal stromal tumor in the stomach: a case report

**DOI:** 10.1186/s13000-018-0741-9

**Published:** 2018-08-25

**Authors:** Jing Chang, Qing Chen, Yuan Jian, Ping Wei, Guang-Zhong Yang, Ying Wang, Xiang-Yang Fang, Qian-Mei Sun

**Affiliations:** 1 Department of Internal Medicine, Beijing Chao-Yang Hospital, Capital Medical University, Gong-Ti South Road 8#, Chao-Yang District, Beijing, China; 2 Department of Hematology, Beijing Chao-Yang Hospital, Capital Medical University, Beijing, China; 3 Department of Pathology, Beijing Chao-Yang Hospital, Capital Medical University, Beijing, China

**Keywords:** Lymphoma, Diffuse large B-cell lymphoma, Gastrointestinal stromal tumor, Splenectomy

## Abstract

**Background:**

Although the primary malignant spleen tumor is relatively rare, lymphoma is the most common splenic malignancy. It can have quite different clinical manifestations that usually lead to relatively poor outcomes, and thus early and accurate diagnosis are of utmost importance.

**Case presentation:**

The present study reports a case of a 67-year-old female with high fever, abnormal spleen (diagnosed by PET/CT) and no obvious lymph node enlargement. After being subjected to splenectomy, the patient was diagnosed with splenic diffuse large B cell lymphoma coexisting with gastrointestinal stromal tumor in the stomach.

**Conclusions:**

To our knowledge, splenic lymphoma accompanied by gastrointestinal stromal tumor in the stomach is rarely reported. This case report discusses the diagnosis and case management of a patient referring to the existing literature.

## Background

Splenic diffuse large B cell lymphoma and gastrointestinal stromal tumor are two different types of cancer, with different histopathology and different immunohistochemical characteristics [1–3]. Splenectomy is a common surgical procedure used to partially or completely remove the spleen [4], that can also be used as diagnostic method for patients with splenic lymphoma [5]. This approach allows for inspection of the abdominal cavity, and detection of abnormal sections. In the present study, the pathologic diagnosis of splenic diffuse large B cell lymphoma coexisting with gastrointestinal stromal tumor in the stomach was based on the spleen excision gastric nodules that were found in patient.

The aim of this work was to discuss whether the use of splenectomy in elderly patients with fever, abnormal spleen, and no other identified symptoms could be useful in making a diagnosis.

## Case presentation

This case study was approved by the Medical Ethics Committee of the Beijing Chao-Yang Hospital, Capital Medical University.

A 67-year-old female, who complained of intermittent fever lasting for 10 months was admitted to the Department of Internal Medicine, Beijing Chao-Yang Hospital, Capital Medical University, Beijing, China. The patient developed fever without an apparent reason (e.g. she did not experience chills before fever), which was the highest during afternoon and evening hours. The patient also complained of urinary urgency and frequency, facial edema, shortness of breath and weakness. She reported no cough, sputum, night sweats or joint pain. Her temperature would usually drop to normal after she would take ibuprofen. Furthermore, the patient visited the local clinic, and was diagnosed with urinary tract infection, which was treated with clindamycin for 4 days. During that period (2 to 3 weeks), the body temperature gradually dropped to normal. No blood tests or other examinations were conducted.

The patient again developed a fever (T_max_ was 38.3 °C), and after visiting the local clinic she was again treated with clindamycin. Nevertheless, this time, the fever didn’t drop following administration of omidazole and levofloxacin for 4 days. Therefore, the patient was admitted to our hospital for further diagnosis and treatment. Physical examination confirmed the following: high body temperature (38.0 °C), blood pressure of 110/70 mmHg, heart rate of 80 Bpm (beats per minute), and respiratory rate of 18 Bpm. Superficial lymph nodes were not palpable.

**Fig. 1:**
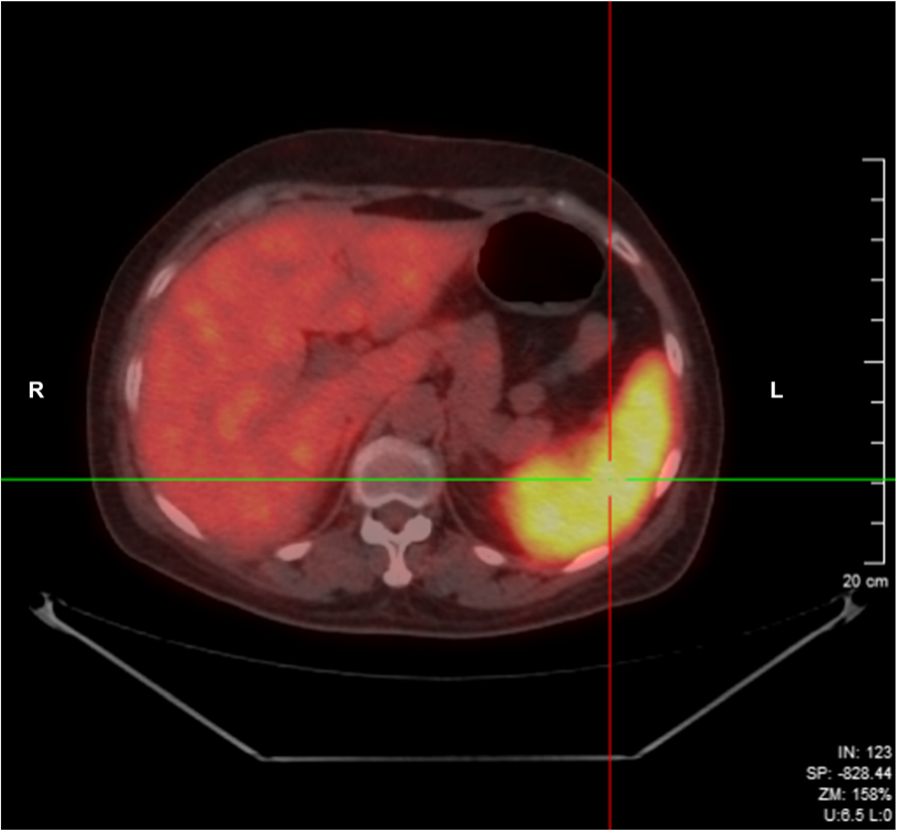
PET-CT of spleen. PET-CT scan revealed spleen abnormality with SUV_max_ 10.2

After admission, the patient’s body temperature fluctuated from 37.3 to 39.0 °C. Blood tests showed white blood cells 5.88*10^9^/L, neutrophil 65.4%, hemoglobin 101 g/L, platelet 293*10^9^/L. Mycoplasma and Chlamydia antibodies, IgM and IgG were both negative. Three sets of blood cultures tested negative. Furthermore, procalcitonin was 0.09 ng/ml, ESR was 110 mm/h, T-SPOT. TB (Oxford Immunotec Global PLC, Abingdon, England) was negative. Blood EB virus and cytomegalovirus antibodies IgG were positive, while IgM were negative. In addition, hepatitis A IgM, hepatitis B antibody, and antigen were all negative. Also, HBV, HCV, HIV and syphilis antibodies all tested negative. Autoantibodies and anti-neutrophil antibodies were negative, as well as tumor markers. Serum biochemistries, including aspartate aminotransferase, alanine aminotransferase, alkaline phosphatase, total bilirubin, serum creatinine, and lactate dehydrogenase were all within normal limits. Cardiac, abdominal and urinary tract ultrasound showed no signs of damage. Enlarged lymph nodes were not detected by chest high-resolution computed tomography (CT) or total abdominal enhanced CT. Nevertheless, abnormal spleen with SUV_max_ 10.2 was observed by PET-CT (Fig. 1). Bone marrow aspiration revealed that lymphocytes accounted for 19.5%, while abnormal suspicious cells for 7% of the total cell count, while no plasma phagocytes were detected. Consequently, splenectomy was conducted to further investigate the organ pathology. During the operation, a small stomach nodule was found, and was immediately resected and analyzed.

**Fig. 2:**
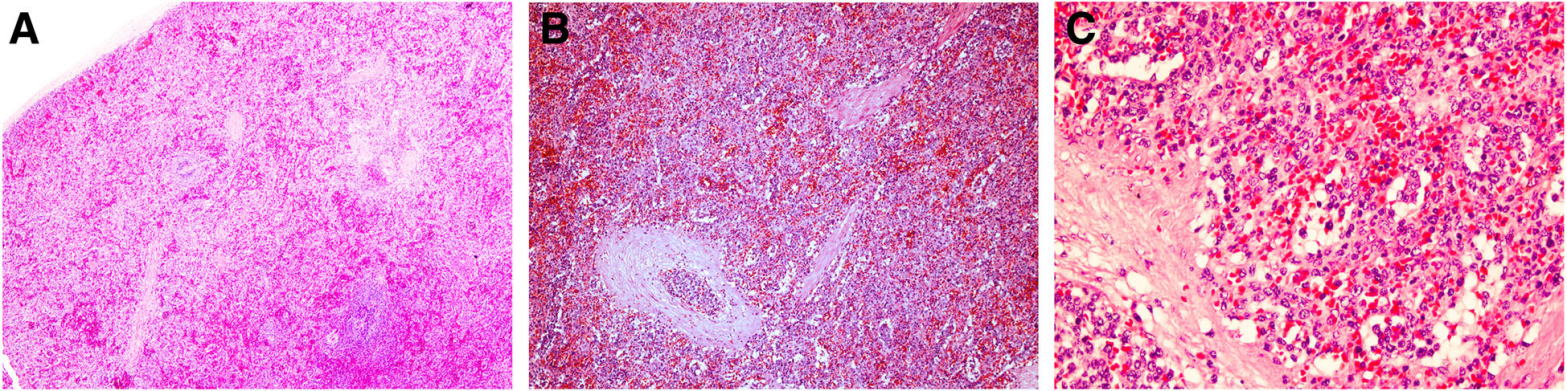
Pathological features of spleen. **a**: HE staining, 4 × 10 magnification. Enlarged pulp sinuses were detected under the microscope. **b**: HE staining, 10 × 10 magnification. The spleen sinuses were filled with the lymphomatous cells. **c**: HE staining, 40 × 10 magnification. The lymphomatous cells were mainly medium-sized with round nuclei and multiple small nucleoli. Mitosis was easily detectable. White pulps were reduced and shrunk

**Fig. 3:**
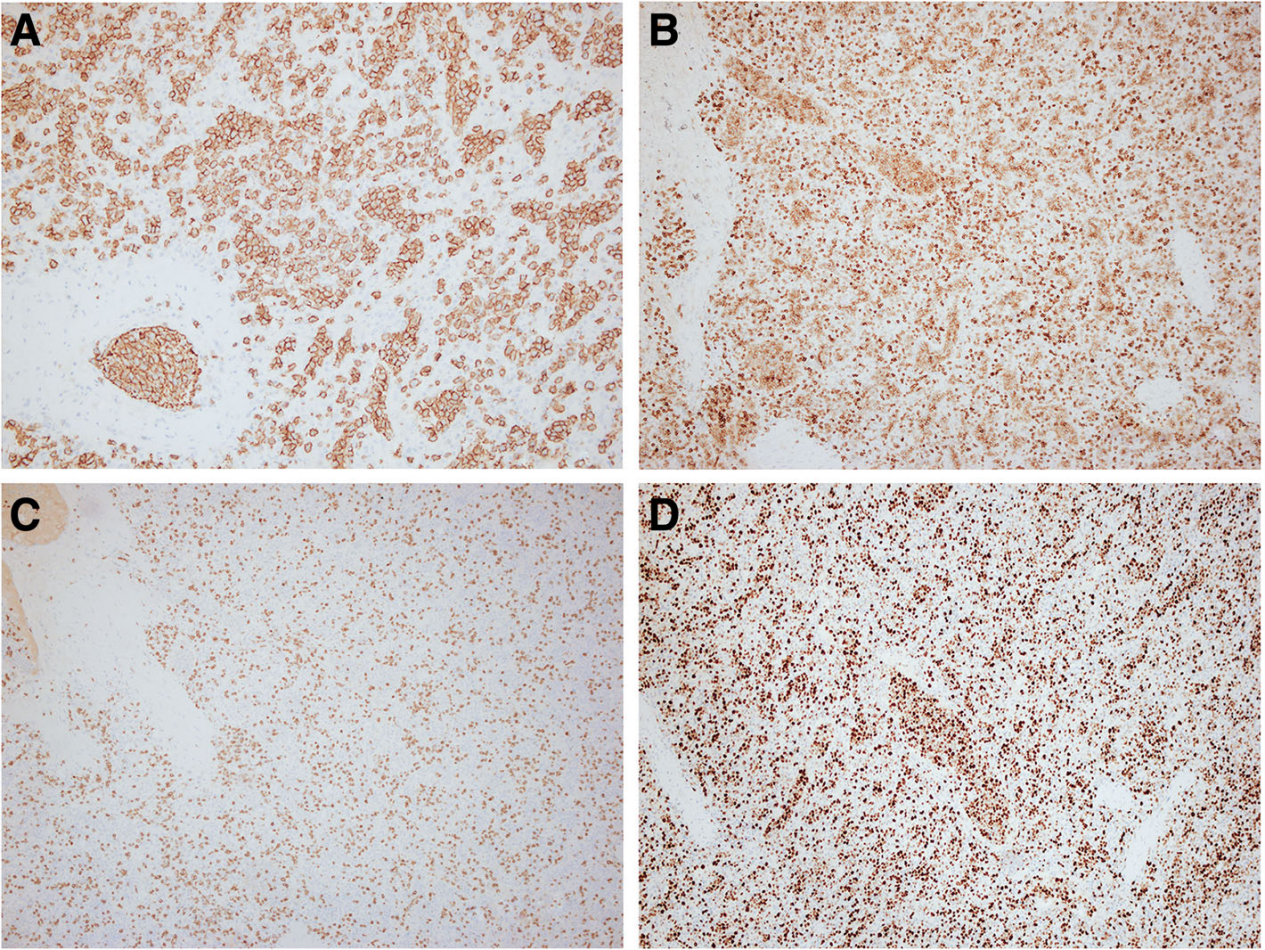
Immunohistochemical features of the spleen. The lymphomatous cells were positive for CD20 (**a**), CD5 (**b**); and negative for CD3 (**c**). Ki – 67 proliferative index was about 80% (**d**)

**Fig. 4:**
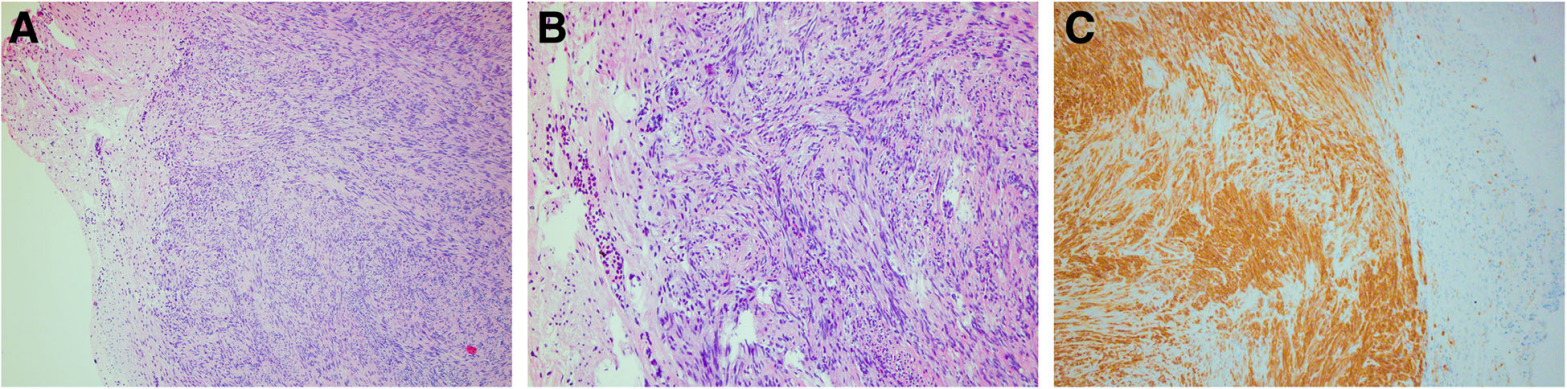
Pathological and immunohistochemical features of gastric nodules. **a**: HE staining, 4 × 10 magnification. Tumor tissue was composed of spindle cells, which were without pleomorphism. **b**: HE staining, 10 × 10 magnification. Lymphomatous cells were of medium size and they were similar to the splenic lymphoma cells in the vessels of the normal gastric tissue. **c**: HE staining, 40 × 10 magnification. Immunohistochemically, the tumor cells were positive for CD117

Pathological examination indicated an obvious enlargement of the spleen (14x12x3.8 cm, weight 410 g), with reddish brown cut surface, while there were no obvious nodules. Following microscope examination, the splenic architecture was normal, the white pulps were reduced and dispersedly distributed, the red pulps were extended, and the splenic sinus was enlarged, containing atypical lymphoid cells. At higher magnification, the atypical cells were large, with 2–3 small nucleolus (Fig. 2). Also, atypical cells were almost limited to venous sinuses. Furthermore, immunohistochemistry data indicated that atypical cells were positive for CD20, CD79a, CD5, Mum-1 and Bcl-2, while they were negative for CD3, CD10, CD23, Bcl-6, CD138, CD163, CD34 and CyclinD1. Kappa and Lambda were positively scattered, while Ki-67 proliferative index was approximately 80% (Fig. 3). Pathological diagnosis was splenic non-Hodgkin B cell lymphoma, i.e. diffuse large B cell lymphoma. Nodule diameter was 0.7 cm, and pathological diagnosis was gastrointestinal stromal tumor (Fig. 4). Furthermore, diffuse large B cell lymphoma cells were found within the vessels around the stomach nodule. The immunohistochemistry results indicated that nodule cells were SMA (−), Desmin (−), Vimentin (+), CD34 (+), CD117 (+), S-100(−), Ki-67-LI (< 5%), Dog-1 (+), CD20 (+). To sum up, the above results supported the diagnosis of splenic diffuse large B cell lymphoma concurrently with gastrointestinal stromal tumor in the stomach.

The patient underwent comprehensive physical examination prior to chemotherapy. Twelve days after splenectomy, she received following R-CHOP chemotherapy regimen: rituximab, 600 mg, day 0; cyclophosphamide, 1.4 g, day 1; doxorubicin hydrochloride liposom, 40 mg, day 1; vincristine, 4 mg, day 1; prednisone, 100 mg, days 1–5. The patient was in remission after 3 treatment courses.

## Discussion

Although rare, lymphoma is the most common splenic malignancy [4–8]. Herein, we introduced a case of an elderly patient who developed a fever but showed no superficial lymph node enlargement or other obvious signs of disease during chest computed tomography (CT) or abdominal CT scan. The patient was further examined by PET/CT which revealed diffuse splenic visceral changes. Consequently, the patient was subjected to splenectomy which confirmed the presence of splenic diffuse large B lymphoma.

According to existing literature, splenic lymphoma can have different clinical manifestations that may simply include abdominal discomfort or spleen enlargement without any other abnormalities. Besides being used for diagnosing the splenic lymphoma, splenectomy is also used to treat splenic lymphoma [4, 7, 9]. It is a feasible surgical method that allows for abdominal exploration of other potential illnesses. Nonetheless, elderly patients undergoing the surgery have poor prognosis. Also, most patients with splenic lymphoma have enlarged spleen, which makes laparoscopic spleen resection harder. There is also a possibility of postoperative complications.

In this case, stomach nodule was found and sectioned through the splenectomy, while gastrointestinal stromal tumor was diagnosed following pathological examinations. Since this patient didn’t present any specific gastrointestinal symptoms, her gastrointestinal stromal tumor could have been misdiagnosed if the splenectomy hadn’t been carried out. Even though case studies on gastrointestinal stromal tumor with lymphoma are very rarely published, the associations between gastrointestinal stromal tumor and other cancers, such as lymphoma have been recently confirmed by a population-based analysis [10, 11]. Consequently, patients with lymphoma might be at higher risk of developing gastrointestinal stromal tumor. Nonetheless, pathogenesis and crosstalk between these two different tumors are still not fully understood and need to be further elucidated. Just like their development and the etiology, which also remain unclear. The present study contributes to the management of lymphoma with gastrointestinal stromal tumor.

## Conclusion

In conclusion, we presented the relatively rare case of splenic lymphoma accompanied by gastrointestinal stromal tumor in the stomach. Pathogenesis and crosstalk between these two different tumors still remain unclear. Splenectomy, which is usually used for diagnosing the splenic lymphoma, can also be used to treat splenic lymphoma. At the same time, it is a feasible surgical method that allows for abdominal exploration of other potential illnesses.

## Abbreviations

Bpm: beats per minute
CT: Computed tomography
ESR: Erythrocyte sedimentation rate
HBV: Hepatitis b virus
HCV: Hepatitis c virus
HE: Hematoxylin-Eosin
HIV: Human immunodeficiency virus
IgG: Immunoglobulin G
IgM: Immunoglobulin M
PET/CT: Positron emission tomography/computed tomography
R-CHOP: Chemotherapy regimen with rituximab, cyclophosphamide, doxorubicin hydrochloride liposom, vincristine and prednisone
SMA: Smooth muscle antigen
SUV: Standardized uptake value
TB: Tuberculosis
